# Work-related stressful events and burnout experienced by residents and specialists in German neurology: prevalence, causes, and coping strategies derived from a nationwide survey

**DOI:** 10.1186/s42466-025-00415-x

**Published:** 2025-07-28

**Authors:** Johannes Heinrich Alexander Piel, Anne-Sophie Biesalski, Robin Wolke, Annette Rogge, Helge Topka, Matthias Klein, Christoph Johannes Ploner, Frank Andres, Daniela Berg

**Affiliations:** 1 Department of Neurology, University Hospital Schleswig-Holstein, Campus Kiel, Kiel, Germany; 2 Department of Neurology, Ruhr-University Bochum, St. Josef Hospital, Bochum, Germany; 3 Department of Neurology, North Sea Clinic Helgoland, Helgoland, Germany; 4 Department of Neurology, Clinical Neurophysiology, Cognitive Neurology and Stroke Unit, Munich Hospital Bogenhausen, Munich, Germany; 5 Department of Neurology, Ludwig Maximilian University of Munich, Großhadern Hospital, Munich, Germany; 6 Department of Neurology, Charité University Medicine Berlin, Berlin, Germany; 7 Department of Neurology and Early Rehabilitation, Kreiskliniken Reutlingen GmbH, Reutlingen, Germany

**Keywords:** Resilience, psychological, Burnout, professional, Internship and residency, Neurology, Occupational stress, Debriefing, Coping strategy, Resilience

## Abstract

**Background:**

Burnout is an increasing challenge and highly prevalent among healthcare professionals. Time-critical emergencies, high workload, the second-victim phenomenon, and moral distress have been identified as key risk factors of burnout. However, measures to mitigate the impact of stressful events have not yet been fully utilized and data in Germany is still limited.

**Methods:**

To address this gap, the Young Neurology section of the German Neurological Society conducted a nationwide survey between October 7 and November 18, 2024, assessing 318 Neurology residents and 175 Neurology specialists. The study examined the frequency of stressful events, risk factors, coping mechanisms, and burnout severity.

**Results:**

Stressful events occurred monthly and most often in emergency rooms, intensive care units, and general wards. Most residents were at risk of burnout and often lacked direct supervision during critical incidents. Common training-independent causes were high patient numbers, the second-victim phenomenon, and poor communication. Knowledge and skill related causes were specific to residents. Burnout was independently correlated to the frequency of stressful events, job satisfaction, institutional factors, age, number of children, and debriefing offer. While job satisfaction was generally good, 30% of participants thought about changing the employer and 10% about leaving Neurology. Dysfunctional coping strategies including the use of alcohol and medication were common and significantly correlated with increased burnout risk. The most relevant mitigation strategies were structured onboarding, debriefing, and improvement of processes.

**Conclusion:**

Our findings confirm high burnout rates, particularly during residency, and highlight the urgent need for targeted intervention.

**Trial registration:**

The study was registered in the German Clinical Trial Register (DRKS-ID DRKS00035214) on 7 October 2024.

**Supplementary Information:**

The online version contains supplementary material available at 10.1186/s42466-025-00415-x.

## Background

Burnout is a state of emotional, physical, and mental exhaustion primarily caused by excessive work-related stress. Its impact on public health is significant, particularly due to reduced clinical hours and workforce turnover, which are estimated to cost U.S. healthcare system approximately $4.6 billion annually [1]. The World Health Organization (WHO) identifies burnout as an individual risk factor for both depression and addiction, distinguishing it from being merely a symptom of depression [2].

Psychological stress among residents is often triggered by unexpected clinical events [1], moral distress [3], or physical assaults from patients or their relatives, as well as an emotional toll of the second victim phenomenon [4–6]. Second victims are healthcare professionals who themselves suffer emotional distress by an unforeseen incident involving a patient, a medical error, or an injury of a patient (first victim) [7, 8]. The combination of high workload and repeated exposure to stressful events can severely contribute to burnout symptoms, especially in healthcare professionals with low resilience [9].

Several mechanisms have been reported to contribute to burnout and secondary diseases in the case of stressful events. The scientific constructs of the second victim phenomenon [6] and moral distress [3] represent the primary subjects of debate in the context of psychophysical stress among healthcare professionals. Far-reaching health consequences, including burnout, coolout, depression, dependency disorders, and even suicidal tendencies, have been reported for both phenomena. Intentions to discontinue practice have been described several times [2]. Coolout or boreout refer to a negative psychological state characterized by low arousal, resulting from of a crisis of meaning at work, crisis of growth, or persistent job boredom [10, 11]. The symptoms of coolout and burnout can be similar and overlap [10].

Neurology, a complex and rapidly evolving medical specialty, presents unique challenges. Residents are frequently exposed to high-stakes emergency situations early in their residency, often within the first year of training [12]. Studies from the United States reveal that 60–75% of neurologists report burnout symptoms, substantially more than the average for other medical specialties [13]. Neurologists have been reported to have one of the lowest satisfaction levels in work-life balance [13], which might influence resilience and burnout susceptibility. Moreover, burnout rates in neurology appear consistent across countries, suggesting inherent risks within the specialty [1].

There is, however, a lack of systematic data on the prevalence and causes of stressful events in German neurology training, as well as on strategies to effectively mitigate them. A recent study involving 109 German neurology residents highlighted the high prevalence of moral distress [14]. Resilience has been recognized as a critical factor in preventing burnout, as demonstrated in German emergency medical services [15], however, the role of personal factors, such as marital status and parenthood, in influencing vulnerability and resilience remains poorly understood.

Understanding factors that contribute to stressful events and identifying strategies to mitigate their impact are essential for fostering successful neurology training. This study aimed to explore the frequency, causes, and resilience factors related to stressful events, as well as to evaluate potential interventions in neurology training. Specifically, the study addresses four key questions:

1. How common are stressful events in neurology training in Germany?
2. What are work-related and personal factors influencing stressful events?
3. Which work-related and personal resources can mitigate the impact of stressful events?
4. Which measures to reduce negative psychological effects are used and experienced as helpful within the group of participants?

## Methods

The survey was conducted between 8 October and 18 November 2024 and collected demographic data, information about the participants’ working environment, the frequency and causes of stressful events, and the coping strategies used. Additional questions were used to investigate who they would have preferred to entrust themselves to and with whom the participants spoke about stressful events. The full questionnaire is listed in the Supplementary Data (Supplementary Table 1). The German Neurological Society (DGN) invited 4,555 residents and 4,529 specialists via E-mail and a call on the official website to participate in the online survey, open to both neurology residents and specialists.

The questionnaire was iteratively developed by members of the DGN committee on neurological emergency medicine, which included neurology head physicians, specialists, and residents with the support of the Young Neurology and DGN executive board. Meaningful items were collected, adapted, and reduced to several items for which a high response rate could be expected.

The survey included the shortened version of the German Burnout Assessment Tool (BAT-12), a validated instrument to evaluate participant’s risk of burnout [16]. The BAT has been validated in German [17], cross-culturally replicated, and demonstrates strong convergent, divergent, and discriminant validity [18]. Two cut-off values have been proposed for BAT-12 total core. This analysis used the more conservative cut-off, which has a reported sensitivity of 72% and specificity of 91% (AUC 0.93) at a false-positive rate of 5%.

To explore factors contributing to the BAT-12 score we chose for a staged analysis procedure. First, to analyze which stressors mentioned contributed to the BAT-12 score we fitted linear multivariate models adjusting for demographic variables including sex, age, family status, hours worked per week, status of residency or specialist, work-place, working hours per week, as well as the frequency of stressful events, whether a debriefing after events was available, and perceived overall job satisfaction. Second, we divided the items into logical categories, summed the numerical values of the responses, normalized them and applied an abstract regression model, as some items may have overlapped to some extent and therefore potentially caused statistical problems associated with multicollinearity.

Statistical analyses were performed using R version 4.4.0 with the use of packages ‘base’, ‘tidyverse’, ‘ggpubr’, ‘ggplot2’ and ‘gtsummary’. For continuous data and ordinal data, Wilcoxon rank-sum tests were applied to compare groups. For categorical data, Fisher’s exact tests were utilized to assess differences between groups. In cases in which multiple answers were possible, we used Fisher’s exact tests category-wise with correction for multiple testing. For paired nominal data we applied McNemar’s test. Kendall’s Tau was used to test correlations involving continuous and ordinal data. If applicable, we applied Holm’s method to correct for multiple testing. Significance was set at an alpha level of 5%.

The study was conducted in accordance with the Declaration of Helsinki and received approval from the Ethics Committee of the Medical Faculty of Kiel University (D 493/24) on 3 May 2024. The study was registered in the German Clinical Trial Register (DRKS-ID DRKS00035214) on 7 October 2024.

## Results

### Socio-demographic data

A total of 493 physicians participated in the study (Table 1). Residents were significantly younger than specialists (*p* < 0.001), less likely to be parents (*p* < 0.001), had less children (*p* = 0.019), and were more frequently employed in hospitals equipped with intensive care units (*p* < 0.001). On average, residents worked 8 h/week more than specialists (*p* < 0.001) and male physicians (mean 48.2 h, 95% confidence interval 46.8–49.7 h) worked 5 h more per week compared to their female or non-binary counterparts (mean 43.2 h, 95% confidence interval 42.0–44.3 h) (*p* < 0.001).

**Table 1:** Demographics of residents and specialists

	Total,	Resident,	Specialist,	*p*-value^*2*^
	N = 493	N = 318^*1*^	N = 175^*1*^	
Year of residency				
1	24 (4.9%)	24 (7.5%)		
2	44 (8.9%)	44 (14%)		
3	51 (10%)	51 (16%)		
4	61 (12%)	61 (19%)		
5	138 (28%)	138 (43%)		
**Age**	34.00 (7.00)	32.00 (6.00)	38.00 (7.00)	< 0.001***
**Sex**				> 0.9
non-binary	1 (0.2%)	1 (0.3%)	0 (0%)	
female	350 (71%)	225 (71%)	125 (71%)	
male	142 (29%)	92 (29%)	50 (29%)	
**Family status**	N = 487	N = 315	N = 172	< 0.001***
With partner and children	222 (45%)	94 (30%)	128 (74%)	
With partner without children	167 (34%)	139 (44%)	28 (16%)	
Single parent	8 (1.6%)	3 (1.0%)	5 (2.9%)	
Single without children	90 (18%)	79 (25%)	11 (6.4%)	
**Number of children**	N = 186	N = 77	N = 109	0.019*
1	58 (31%)	32 (42%)	26 (24%)	
2	101 (54%)	39 (51%)	62 (57%)	
3	24 (13%)	5 (6.5%)	19 (17%)	
4	3 (1.6%)	1 (1.3%)	2 (1.8%)	
**Work place**	N = 492	N = 317	N = 175	< 0.001***
University hospital	122 (25%)	92 (29%)	30 (17%)	
Tertiary care	119 (24%)	76 (24%)	43 (25%)	
Primary care, ER + ICU+	151 (31%)	105 (33%)	46 (26%)	
Primary care, ER + ICU-	18 (3.7%)	9 (2.8%)	9 (5.1%)	
Primary care, ER-ICU-	27 (5.5%)	16 (5.0%)	11 (6.3%)	
Practices / Medical centers	47 (9.6%)	19 (6.0%)	28 (16%)	
Non-clinical (e.g. scientific)	8 (1.6%)	0 (0%)	8 (4.6%)	
**Working hours per week**	45.00 (10.00)	48.00 (13.75)	40.00 (16.00)	< 0.001***

^*1*^ *n (%); Median (IQR)*, ^*2*^*Fisher’s exact test; Wilcoxon rank sum test*

*ER: Emergency room*,* ICU: Intensive care unit. +: available*,* -: not available*, * p < 0.05, ** p < 0.01, *** p < 0.001 for differences between residents and specialists

### Frequency and causes of stressful events

51% of the participants reported monthly or more frequent stressful events with residents reporting a higher frequency (*p* < 0.001) (Table 2). Most stressful events were reported in the emergency room (391), intensive care unit (ICU) (251), and general wards (213). Stressful events were less frequently in outpatient clinics (43), prehospital settings (36), medical care centers (19), and intervention and diagnostic units (14). Under the option to enter free-texts, stroke unit/intermediate care (IMC) wards (32), psychiatric wards (4), and early rehabilitation wards (5) were explicitly specified. More specialists reported stressful events in the ICU compared to residents (*p* < 0.01).

**Table 2:** Frequency and circumstances of stressful events, as well as burnout-assessment-Tool-12 (BAT-12) scores for neurology residents and specialists

	Total,	Resident,	Specialist,	*p*-value^*2*^
	N = 493^*1*^	N = 318^*1*^	N = 175^*1*^	
**Frequency of stressful events**				< 0.001***
Daily	8 (2%)	6 (2%)	2 (1%)	
Several times per week	36 (7%)	24 (8%)	12 (7%)	
Once per week	32 (6%)	25 (8%)	7 (4%)	
A few times per month	103 (21%)	74 (23%)	29 (17%)	
Once per month	74 (15%)	56 (18%)	18 (10%)	
A few times per year	230 (47%)	128 (40%)	102 (58%)	
Never	10 (2%)	5 (2%)	5 (3%)	
**Location of stressful events**	N = 461	N = 296	N = 165	**p-value** ^**3,4**^
Prehospital care	36 (8%)	25 (8%)	11 (6%)	1
Practices / Medical center	19 (4%)	10 (3%)	9 (5%)	1
Outpatient clinic	43 (9%)	26 (9%)	17 (10%)	1
General ward	213 (46%)	133 (45%)	80 (48%)	1
Emergency room	391 (85%)	256 (86%)	135 (82%)	1
Intensive care	251 (54%)	144 (49%)	107 (65%)	< 0.01**
Interventions / diagnostics	14 (3%)	8 (3%)	6 (4%)	1
*Stroke / Intermediate care*†	32 (7%)	22 (7%)	10 (6%)	1
*Rehabilitation ward*†	5 (1%)	3 (1%)	2 (1%)	1
*Consultation service*†	2 (0.4%)	0 (0%)	2 (1%)	1
*Psychiatric ward*†	4 (0.9%)	2 (1%)	2 (1%)	1
**Situation during stressful events**	N = 458	N = 294	N = 164	**p-value** ^**3,4**^
Alone	314 (69%)	194 (66%)	120 (73%)	0.47
Peer on site	164 (36%)	102 (35%)	62 (38%)	1
Supervised remotely	342 (75%)	232 (79%)	110 (67%)	< 0.05*
Supervised on site	107 (23%)	69 (23%)	38 (23%)	1
Been only observer	37 (8%)	24 (8%)	13 (8%)	1
**Burnout-assessment-Tool-12 **‡	N = 408	N = 267	N = 141	
Total-core	2.50 (0.75)	2.58 (0.83)⁑	2.33 (0.83)	< 0.001***
Exhaustion	3.33 (1.33)⁑	3.33 (1.00)⁑	3.00 (1.33)	< 0.001***
Mental distance	2.33 (1.33)⁑	2.33 (1.33)⁑	2.00 (1.33)	0.002**
Cognitive impairment	2.33 (0.67)	2.33 (0.67)	2.00 (1.00)	0.025*
Emotional impairment	2.00 (1.00)	2.00 (1.33)	1.67 (0.67)	0.012*

^*1*^*n (%); Median (IQR)*, ^*2*^*Wilcoxon rank sum test for ordinal data*, ^*3*^*Fisher’s exact test; Wilcoxon rank sum test*, ^*4*^*Correction for multiple testing using holm’s method*, †* Reported in free-text option*, ‡*Likert scale (1–5)*, ⁑ *at risk of burnout*, * p < 0.05, ** p < 0.01, *** p < 0.001 for differences between residents and specialists

**Fig. 1:**
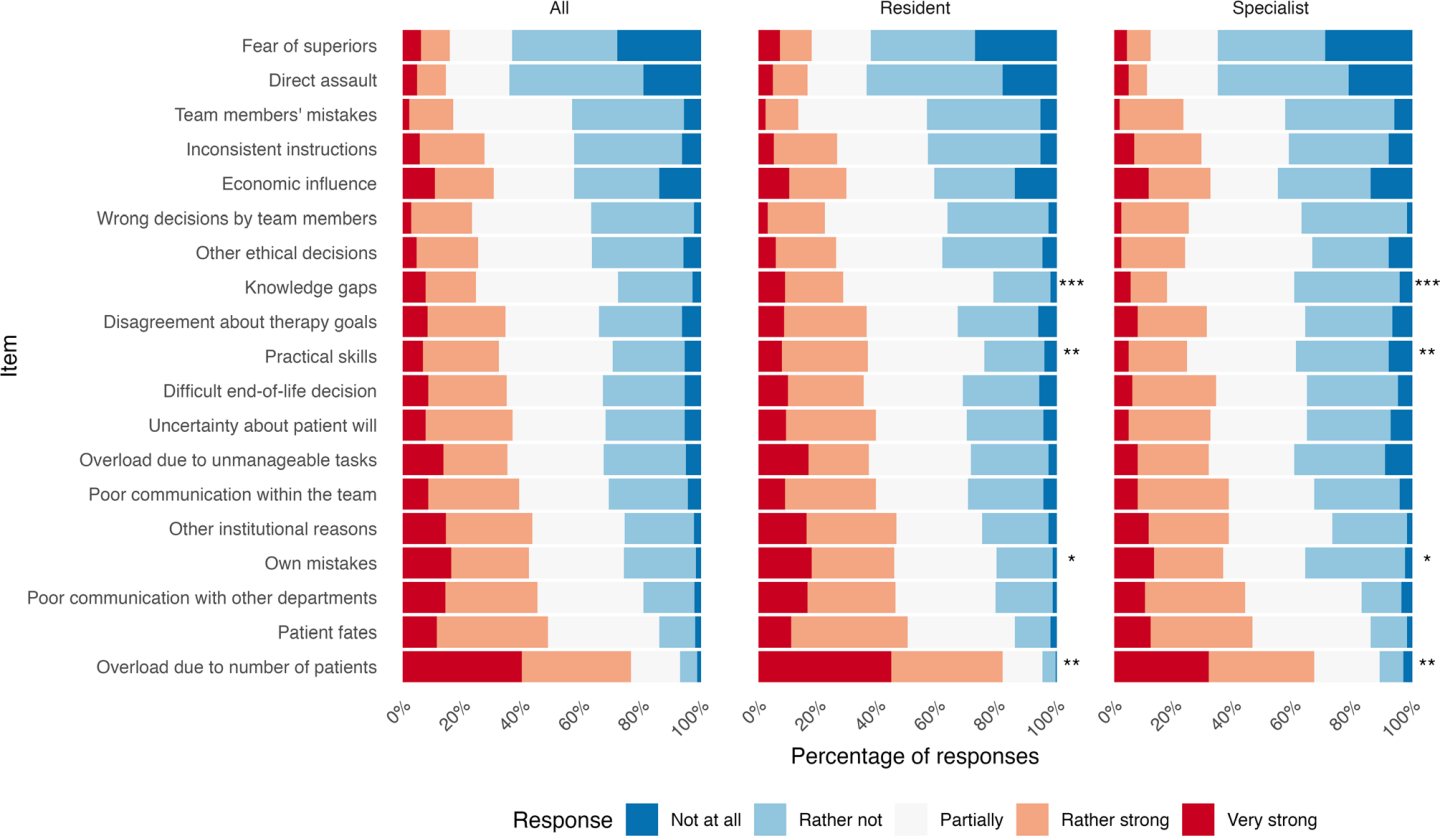
Causes of stressful events, ranked by residents and specialists. (* p < 0.05, ** p < 0.01, *** p < 0.001 for differences between residents and specialists,* Wilcoxon rank sum test for ordinal data*,* Correction for multiple testing using Holm’s method)*

Leading causes of stressful events were work overload due to patient load, second victim phenomenon, poor communication with other departments, mistakes, and organizational factors (Fig. 1). Least influencing were fear of superiors, physical assault, and mistakes made by team members. Residents significantly more often cited knowledge gaps, skill deficiencies, personal mistakes, and overload from patient numbers as causes of stressful events. Knowledge gaps and skill deficiencies negatively correlated with the year of residency (τ = -0.128, *p* = 0.010 and τ = -0.161, *p* = 0.001 respectively).

39% reported the presence of peers during stressful events. On-site supervision was available in 23% of stressful events. Residents were significantly more often remotely supervised (e.g., having the ability to call superiors) than specialists during stressful events (*p* < 0.05).

### Preparation for and mitigation of stressful events

63% reported they had not been adequately prepared to handle stressful events. 4% of the respondents have been prepared before starting their studies, 17% during their studies and 16% during residency. 17% prepared themselves independently of the profession. We found no statistical differences between residents and specialists in training to handle stressful events (Supplementary Table 4).

Recreational activities outside of the hospital (84%), including sports (68%), music (38%) and hobbies (38%) were the most frequently used coping strategies. 32% stated they immersed themselves even more in their work. 20% of respondents drank alcohol after stressful events, 9% took medication. Some colleagues utilized external supervision (11%) after stressful events, specialists significantly more frequently (*p* = 0.01). Various coping strategies mentioned in free text form included sleep, meditation, relaxation techniques, faith, crying, psychotherapy, and even resentment.

**Fig. 2:**
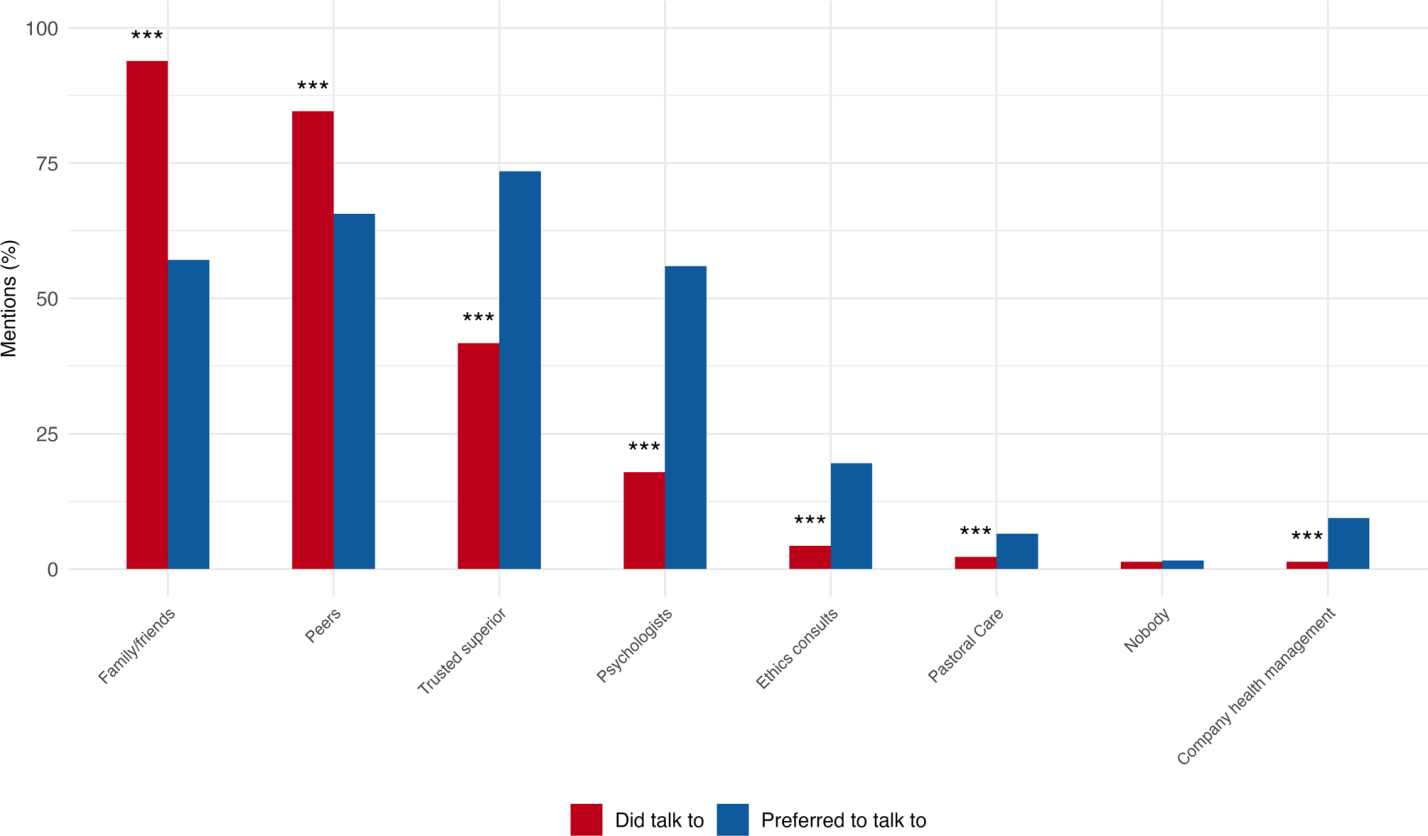
Comparison of actual vs preferred advice connections after stressful events (in percent), N = 494. (Percentages of mentioned categories are based on overall number of mentions. Multiple answers per individual were possible for both ‘Did talk to’ and ‘Preferred to talk to’. *** p < 0.001 for differences between residents and specialists,* McNemar Test for paired nominal data*,* correction for multiple comparisons using Holm’s method)*

Residents and specialists predominantly talked to family and friends (94%) and peers (85%) after stressful events (Fig. 2). 42% of respondents discussed such events with a trusted superior. Specialists were significantly more likely to consult ethics counsellors (Likelihood ratio = 8.58, *p* = 0.003). Respondents significantly more often preferred to talk to trusted superiors, psychologists, ethics consults, pastoral care, and company health management and significantly less often preferred to talk with family, friends, and peers (*p* < 0.001). A detailed visualization which participants would have preferred to speak to which group is presented in the Supplementary Data (Supplementary Fig. 1).

**Fig. 3:**
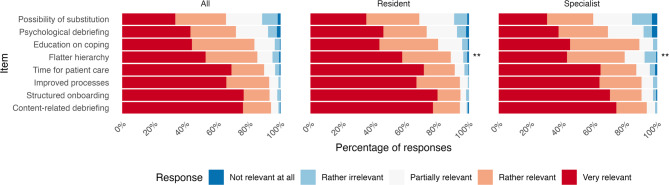
Relevance of potential measures to mitigate stressful events, ranked by residents and specialists. (*p < 0,*05*, ** p < 0.01, *** p < 0.001 for differences between residents and specialists,* Wilcoxon rank sum test for ordinal data*,* Correction for multiple testing using Holm’s method)*

Residents and specialists reported structured onboarding and content-specific debriefing to be the most relevant measures to mitigate the effects of stressful events. Residents ranked flatter hierarchy significantly higher. Education on coping, psychological debriefing, possibility of substitution, and improvement of processes were lesser ranked, but all measures received high relevance scores (Fig. 3).

90% of residents and 77% of specialists reported that internal debriefing procedures were either nonexistent or unknown to them (Supplementary Table 2).

### Stressful events and risk of burnout

The median BAT-12 total-core, as well as the exhaustion and mental distance subscales, showed risk of burnout for residents (cut-offs: 2.54–2.95, 3.17–3.50 and 2.17–3.16 respectively). 27% of the residents were categorized as at risk of burnout (cut-off: 2.54–2.95) and 26% as likely having burnout (cut-off: ≥ 2.96). The median values for cognitive impairment and emotional impairment subscales remained within normal limits. Neurology residents scored significantly higher in the BAT-12 total-core and in all subscales than neurology specialists (Table 2). The mean values for the specialists were within normal limits.

**Fig. 4:**
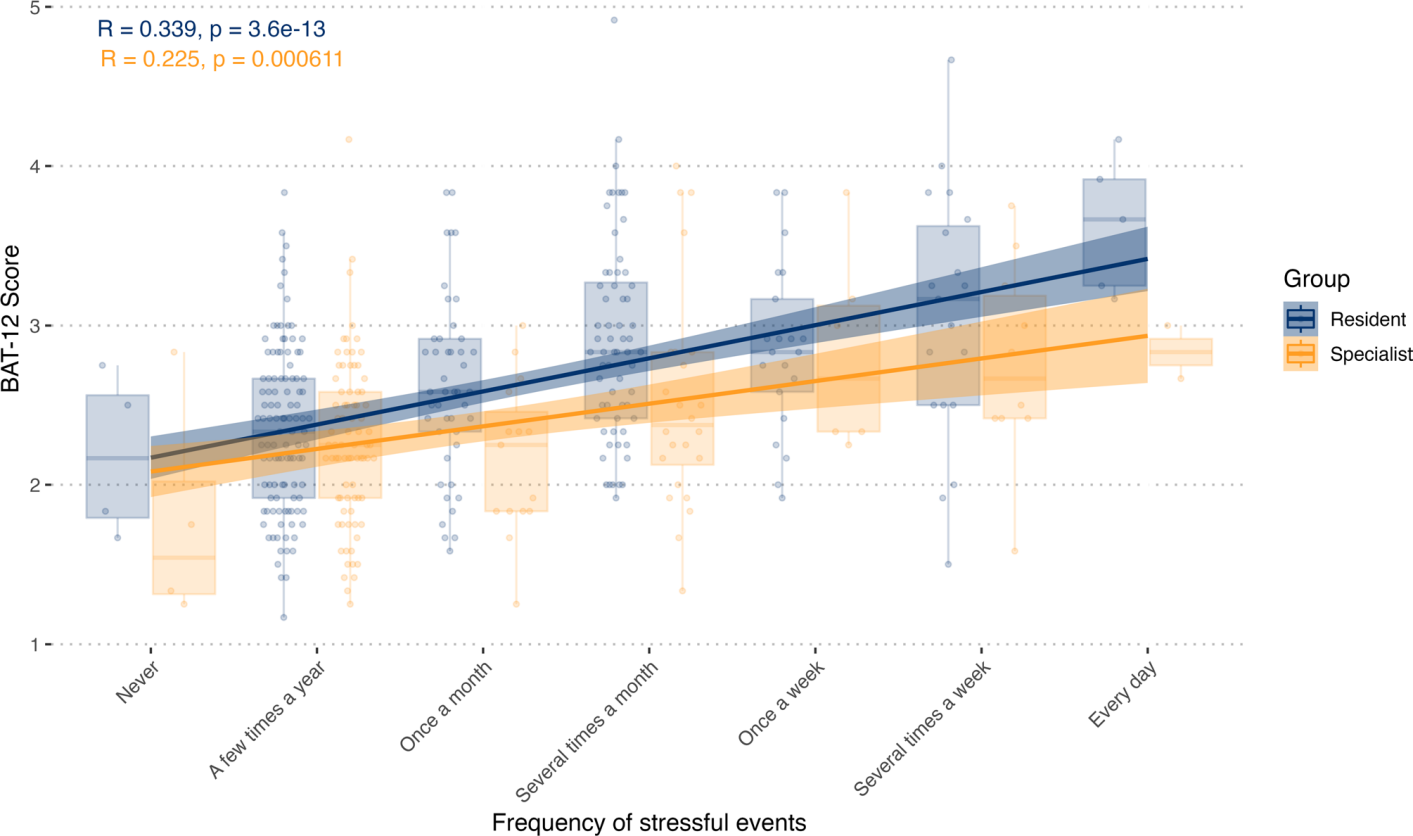
Correlation of the frequency of stressful events and the BAT-12

BAT-12 total-core significantly correlated with the frequency of stressful events (ρ = 0.420, *p* < 0.001) (Fig. 4), with a stronger effect in residents. Higher BAT-12 values significantly correlated with working hours (τ = 0.099, *p* = 0.005), the hospital type (F = 2.196, *p* = 0.043, Supplementary Fig. 4), and negatively with job satisfaction (τ = -0.332, *p* < 0.001, Supplementary Fig. 5). Job satisfaction in different workplaces is shown in Supplementary Fig. 6. The correlation between working hours and BAT-12 was stronger in females and non-binary individuals (τ = 0.140, *p* < 0.001) than in males (τ = -0.003, *p* = 0.96) (Supplementary Fig. 2). Neurologists using alcohol (*p* = 0.029) or medication (*p* = 0.004) after stressful events had significantly higher BAT-12 scores, while other coping strategies showed no significant association with burnout. Burnout did not differ by sex.

A multiple linear regression identified a higher frequency of stressful events, low job satisfaction, institutional factors, age, lower number of children, and absence of debriefing offers as independent predictors of burnout (Table 3). Grouped variables and multicollinearity analysis are explained in the Supplementary Data (Supplementary Tables 3 and Supplementary Fig. 3).

**Table 3:** Multivariable linear regression model of the association between potential causes of stressful events and burnout symptoms

Variable		Coefficient	*p*-Value
(Intercept)		0.5504	0.103
**Age**		0.0152	0.030*
**Sex**			
	Female	Reference	
	Male	-0.0223	0.735
**Training Status**			
	Resident	Reference	
	Specialist	-0.0585	0.449
**Relationship Status**		Reference	
	Single		
	With partner	-0.0013	0.986
**Number of children**		-0.0786	0.046*
**Working hours**		0.0004	0.905
**Frequency of stressful events**		0.1097	< 0.001***
**Debriefing available**			
	No	Reference	
	Yes	-0.1800	0.037*
**Job satisfaction**		-0.1104	< 0.001***
**Workplace**			
	University clinic	Reference	
	Tertiary care	0.1086	0.172
	Primary care, ER + ICU+	0.1295	0.092
	Primary care, ER + ICU-	-0.2223	0.130
	Primary care, ER-ICU-	0.0300	0.825
	Practices / Medical centers	-0.2217	0.059
	Non-clinical (e.g.scientific)	0.1150	0.765
**Ethical Factors**		0.0054	0.549
**Team Communication**		0.0046	0.703
**Institutional Factors**		0.0431	< 0.001***
**Mistakes**		0.0055	0.733
**Professional Skills**		0.0090	0.639
**Direct assault**		0.0374	0.180

### Job satisfaction and job change

Job satisfaction was generally good, but significantly lower in residents than specialists (*p* < 0.001). Among residents, 52% agreed or somewhat agreed that they were satisfied with their job, while 26% disagreed or somewhat disagreed, compared to 70% and 16% in specialists. Significantly more residents considered changing jobs (*p* = 0.021). Over 30% considered changing employers, 10% (12% of residents and 6% of specialists) considered leaving neurology, and 2% considered leaving medicine entirely within the next 12 months. Higher BAT-12 values were associated to the desire to leave medicine, neurology, or their employer (F = 20.11, *p* < 0.001). Low job satisfaction significantly correlated with the frequency of stressful events (*n* = 429, Likelihood ratio 53.04, *p* < 0.001).

## Discussion

Our survey highlights the significant impact of stressful events on Neurology residents’ and specialists’ mental health, linking them to distress, low job satisfaction, and burnout, particularly among residents. We identified possible causes as well as ways to mitigate distress. Despite overall high job satisfaction, many physicians consider changing employers or the specialty. Acute medical scenarios, high patient load, and the second victim phenomenon, were key stressors. Residents, in particular, reported greater distress due to skill and knowledge gaps, emphasising the need for structured onboarding and regular debriefings.

### Individual factors

Workload was a key predictor of burnout. While reduced working hours lower burnout risk [19, 20] and part-time work mitigates work-life-conflicts [21], our data suggest that burnout is mediated by job satisfaction and frequency of stressful events, rather than hours worked alone [21, 22]. Thus, interventions should address both working time models and workplace stressors. Optimizing self-organization and institutional processes, such as reducing documentation redundancy and task shifting towards medical activities, could free up further resources needed in an environment of continued increase in patient load and expected shortage of physicians [23].

Women, despite working fewer hours on average, were more affected by long working hours than men, likely due to the double burden of childcare and professional responsibilities [24]. Conversely, having a partner and children was linked to greater resilience and lower burnout rates. Relationships have been shown to reduce burnout risk [25], though the impact of parenthood on psychological well-being remains complex, with varying effects reported [26–28].

### Institutional factors

We have shown neurology physicians are subject to partially mitigable stressful events and residency to be a particularly vulnerable period. Knowledge gaps, skill deficiencies, and high patient load contributed to distress and burnout during residency. Structured onboarding and targeted preparation were the most frequently requested measures to alleviate distress.

Our data support the well-established theme that distress peaks during residency, driven by perceived knowledge gaps, newly assumed responsibilities, and the need to manage uncertainty [29–31]. Neurology physicians have been reported to exhibit lower resilience to stress compared to those in other specialties, such as emergency medicine and neurosurgery [32], which may partly explain their high distress in emergency situations. In contrast, a meta-analysis of 4,664 residents found an overall burnout prevalence of 35.7% across specialties and reported neurology with a low burnout rate of 18.1% [33]. However, this low burnout rate resulted from a single Greek study involving only 116 neurologists, limiting the generalizability of the finding. A more recent meta-analysis reported that 65.9% of neurologists experience burnout, with prevalence rates ranging widely from 18.1 to 94% across six countries [1]. Cultural, religious, and societal factors, along with differences in work ethics, training programs, and living standards, may account for these disparities and underscore the importance of conducting burnout research within specific national contexts.

High levels of burnout in young professionals have also been reported in other professions with high workloads and not all causes are inherent to medicine [34]. However, when considering targeted approaches, we provide evidence of a particular need for thorough training in emergency medicine in neurology.

The second victim phenomenon has gained recognition, with over 50% of internal medicine physicians in German-speaking countries reporting experience with it [4, 5]. Our data align with these findings when considering errors made by oneself and team members. Further research using established second victim questionnaires is warranted to provide deeper insights.

Several of the items surveyed can be harmonized with the scientific construct of moral distress, as seen in a recent study [14] where neurology residents identified the inability to invest sufficient time in patient or family consultation as their most distressing experience.

Neurology physicians employed a range of coping strategies, from recreational activities to increased work engagement. However, maladaptive coping mechanisms, such as alcohol or medication use, were alarmingly prevalent, particularly among those with high burnout rates. Unhealthy behaviors like poor diet, alcohol consumption, and physical inactivity may worsen psychological well-being and health, contributing to a vicious cycle [35, 36].

### Stress management and debriefing

Structured stress management approaches are well established in health care [37], particularly emergency medicine, public safety, and aviation [38]. Measures such as clinical scenario training, improved supervision, and structured debriefings enhance resilience and job satisfaction [39]. Despite the widespread use of Critical Incident Stress Management (CISM), a structured debriefing method, in public safety and aviation [38], implementation in medicine has been criticized [40, 41] and the WHO has highlighted the need for further research [40, 42].

Although debriefing has faced criticism [40], a recent review suggests multifaceted interventions, combining educational and clinical interventions, as well as debriefings, may help mitigate the second victim phenomenon [43]. Further research is warranted to determine which debriefing and intervention methods are safe and most effective.

We identified a substantial gap between the need for professional debriefing support and reliance on informal networks. Many participants desired discussions with trusted superiors or psychologists, yet barriers such as limited availability, stigma, misconceptions, and time constraints may be hindering. A cultural shift from a person-focused blame culture towards a process-oriented just culture is essential for improving resilience and patient safety [44, 45]. In a just culture, mistakes are acknowledged to result from both individual actions and systemic weaknesses. Open discussions of adverse events and errors are encouraged, not to assign blame, but to identify opportunities for learning and process improvement [44]. Lower burnout rates among those aware of debriefing opportunities further support this.

## Conclusions

Our study highlights the challenges faced by neurology physicians and potential mitigation strategies. Structured onboarding, content-specific debriefings, and organizational optimizations to minimize stress, especially in emergency medicine, should be prioritized. Further studies comparing specialties could clarify which stressors are inherent to medicine and which are specialty-specific.

### Limitations

We invited all physicians who were members of the DGN at the time of the survey via an announcement in the DGN E-mail newsletter. Of the 9,084 physicians who were members of the DGN as of August 30, 2024, and were therefore invited to participate, 493 (5.43%) completed the survey– comprising 6.98% of neurology residents and 3.86% of neurology specialists.

While the low response rate may be attributed to broad invitation extended to all DGN members, the absolute number of participants is substantial compared to other European studies on Burnout among neurologists, which reported 116 participants in Greece and 188 in Spain [46, 47]. However, it remains modest when compared to larger studies conducted in the United States, such as one with 1,671 participants [19]. The sample is not representative, as a selection bias cannot be ruled out due to recruiting through a scientific society. This may also account for the higher proportion of residents in advanced stages of training. Physicians experiencing burnout may be more willing to participate in a study on burnout and resilience, which could contribute to a selection bias. Moreover, mistakes and exposure to stressful events may be perceived by physicians as signs of weakness. As a result, some physicians may have avoided acknowledging their own distress or chosen not to participate in this study, potentially leading to a self-selection bias.

The questionnaire contained “ICU” as an answer option, intended to encompass all monitoring wards, however the wording did not clearly include stroke or IMC wards. In free-text responses, stroke and IMC wards were explicitly mentioned. Like all free-text responses, they were consequently listed as a separate item. The number of stressful events in intensive care wards may therefore have been overestimated and those in stroke / IMC wards may have been underestimated in our sample.

Apart from the validated BAT-12 score, our survey was developed within a working group and refined iteratively, but it has not been previously validated for measuring stressful event frequency and severity.

We observed a floor effect in the frequency of stressful events. A more detailed analysis of physicians who rarely experienced stress might have influenced the correlation strength with job satisfaction, though overall, the correlation was robust.

## Electronic supplementary material

Below is the link to the electronic supplementary material.

Supplementary Material 1

## Data Availability

The datasets used and/or analysed during the current study are available from the corresponding author on reasonable request but restrictions may apply on data that may compromise anonymity of participants. The data are not publicly available due to privacy or ethical restrictions.

## Abbreviations

AUC: Area under curve
BAT: Burnout Assessment Tool
BAT-12: Shortened version of the German Burnout Assessment Tool
CISM: Critical Incident Stress Management
DGN: German Neurological Society
ICU: Intensive care unit
IMC: Intermediate care
WHO: World Health Organization
