# Supplementary material for: Work-related stressful events and burnout experienced by residents and specialists in German neurology: prevalence, causes, and coping strategies derived from a nationwide survey
Source: Neurol Res Pract. 2025 Jul 28;7(1):52. doi: 10.1186/s42466-025-00415-x (PMC12306127; doi:10.1186/s42466-025-00415-x)

**Supplementary Data**

**Supplementary Table 1.** The translated consensus-based questionnaire. Additionally, the German short version of the burnout assessment tool (BAT-12) was included in the questionnaire.

| Item | Answers | Input option |
| --- | --- | --- |
| Training status | - Resident (1^st^ year) - Resident (2^nd^ year) - Resident (3^rd^ year) - Resident (4^th^ year) - Resident (from 5^th^ year) - Specialist - Doctor with supervisory function | Single choice |
| Age |  | Number |
| Sex | - Male - Female - Diverse | Single choice |
| I have ___ kids |  | Number |
| I primarily work | - Non-clinical (e.g. science) - In a practice / medical center - In a primary/standard care hospital without an emergency department - In a primary/standard care hospital with an emergency department without an intensive care unit/respiratory beds - In a primary/standard care hospital with an emergency department and intensive care unit/respiratory beds - In a non-university maximum care hospital - In a university hospital providing maximum care | Single choice |
| My average actual weekly working hours are ___ |  | Number |
| In the course of my medical work, I have already experienced events that have put me under great psychological strain | - Never - At least a few times a year - At least once a month - A few times a month - Once a week - Several times a week - Every day | Single choice |
| Events that put me under psychological strain me were | - Direct threat/assault of patients or relatives - Patient’s fates - Knowledge gaps - Lack of routine in practical skills - Own wrong decisions - Wrong decisions made by team members - Own (near) mistakes - (Near) mistakes made by team members - Ambiguities about the patient's presumed wishes - Disagreement between the practitioners about the treatment goal - Difficult end-of-life decisions - Other ethical decisions - Poor communication within the team - Difficult communication with other departments - Different treatment announcements within the team - Fear of superiors - Influence of economic factors on treatment - Overload due to the number of patients - Overload due to taking over activities that are not mastered - Other institutional difficulties (e.g. in equipment or organisational structures) | Likert scale for each item |
| I have encountered stressful events in the following functions in particular | - Rescue service - Practice - Outpatient clinic - General ward - Emergency room - Intensive care unit - Operating theatre / intervention function - Other (please specify) | Multiple choice |
| During stressful events ___ | - I was responsible on my own - I was responsible with colleagues at the same level of training - A person responsible for supervision was available for me by telephone - A person responsible for supervision was available for me on site - I were only involved as an observer | Multiple choice |
| Have you been prepared to deal with stressful events? | - I have not yet been prepared for stressful events - I have been trained on this topic before my academic training curriculum (e.g. school) - I have received training on this topic during my studies - I have received further training on this topic after my studies, during further training - I have received further training on this topic regardless of my profession or training | Multiple choice |
| I have already used the following coping mechanisms: | - Alcohol - Medication - Sports - Music - Other hobbies - Distraction outside the clinic, e.g. with friends - External supervision - Deepening in the work - Other (please specify) | Multiple choice |
| I have talked about stressful events with ___ so far | - Nobody - With friends/family (private) - With peers (e.g. colleagues, professionally) - With a trusted person (e.g. superior doctor, professional) - With psychologically trained persons - With a clinical ethics counsellor - With the company health management team - With pastoral care - Other (please specify) | Multiple choice |
| I generally wish I could talk to ___ about stressful events | - Nobody - With friends/family (private) - With peers (e.g. colleagues, professionally) - With a trusted person (e.g. superior doctor, professional) - With psychologically trained persons - With a clinical ethics counsellor - With the company health management team - With pastoral care - Other (please specify) | Multiple choice |
| In our department, we talk openly with superiors about how to deal with stressful events | - Agree - Rather agree - I neither agree nor disagree - Rather disagree - Disagree | Single choice (Likert) |
| In our department, there are opportunities for Critical Incident Stress Management (CISM), stress management after stressful events (SbE), debriefing or psychological debriefings | - No - I am not informed about this - We have an internal hospital procedure (e.g. regular internal debriefings), but I do not use it yet - We have an organised structure (e.g. CISM/SbE), but I do not use it yet - We have an internal clinic procedure (e.g. regular internal debriefings) and I have made use of it - We have an organised structure (e.g. CISM/SbE) and I have made use of it | Single choice |
| I consider the following measures to be particularly relevant for coping with stressful events | - Clarification and learning of coping mechanisms - Content debriefing after stressful events - Psychological debriefing after stressful events - Space to process after stressful events (e.g. possibility of substitution) - Flatter hierarchy / better relationship with superiors - Structured preparation in terms of content before taking on tasks (e.g. induction curricula in neurological emergency medicine) - More time in patient care - Improved processes (e.g. organisational structure in interdisciplinary/central emergency departments) - Other (please specify) | Multiple choice |
| After stressful events, there was something that helped me in particular: |  | Open answer |
| I am generally satisfied with my current job | - Agree - Rather agree - I neither agree nor disagree - Rather disagree - Disagree | Single choice (Likert) |
| I am considering within the next year | - Not to change my employment - To change the activity within the employer - To change the employer - To leave the speciality of neurology - To leave medicine altogether | Single choice |

**Supplementary Figure 1.** Sankey diagram of whom neurology residents and specialists talked to and who they would have preferred to talk to after stressful events. Neurologists who discussed stressful events with family and friends were significantly more likely to express a desire to continue talking with friends (p < 0.001) and peers (p = 0.017), but were less inclined to seek support from company health management (p = 0.042). Talking to peers strongly correlated with the desire discuss stressful events with peers (p < 0.001), trusted superiors (p < 0.001) and ethics counsellors (p = 0.023). Similarly, those who spoke to trusted superiors or psychologists expressed a significant desire to continue these discussions (p < 0.001), but neurologists who consulted psychologists also significantly sought to discuss stressful events with company health management (p = 0.001). Neurologists who consulted ethics counsellors or company health management were also significantly more likely to seek ongoing engagement with these professions (p < 0.001). *(Percentages of mentioned categories are based on overall number of mentions. Multiple answers per individual were possible for both 'Did talk to' and 'Preferred to talk to'. *** p < 0.001, McNemar Test for paired nominal data, correction for multiple comparisons using Holm's method.)*


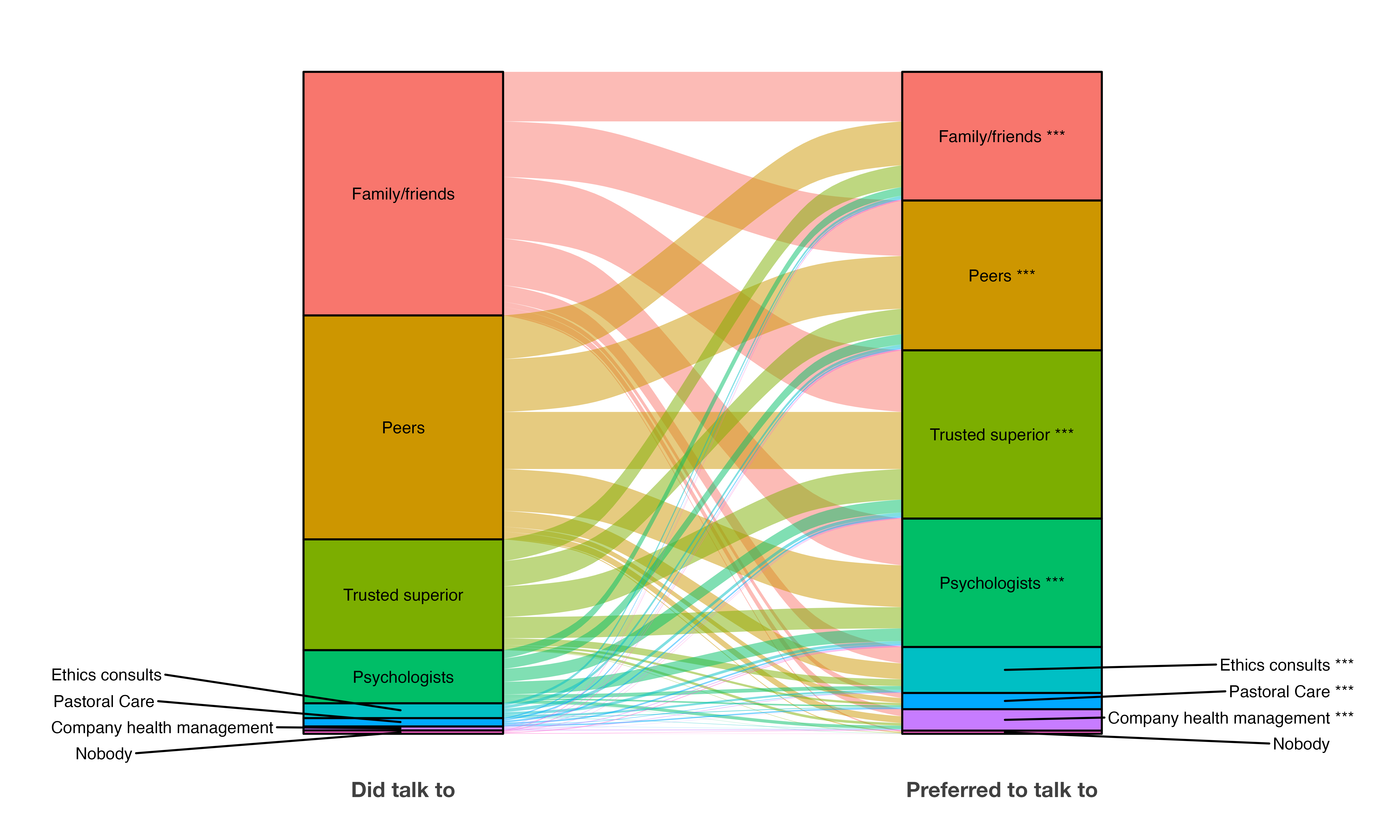


**Supplementary Figure 2.** The correlation of working hours and BAT-12 score is sex-dependent. While females show a positive correlation of working hours / week with BAT-12, males did not exhibit this correlation.


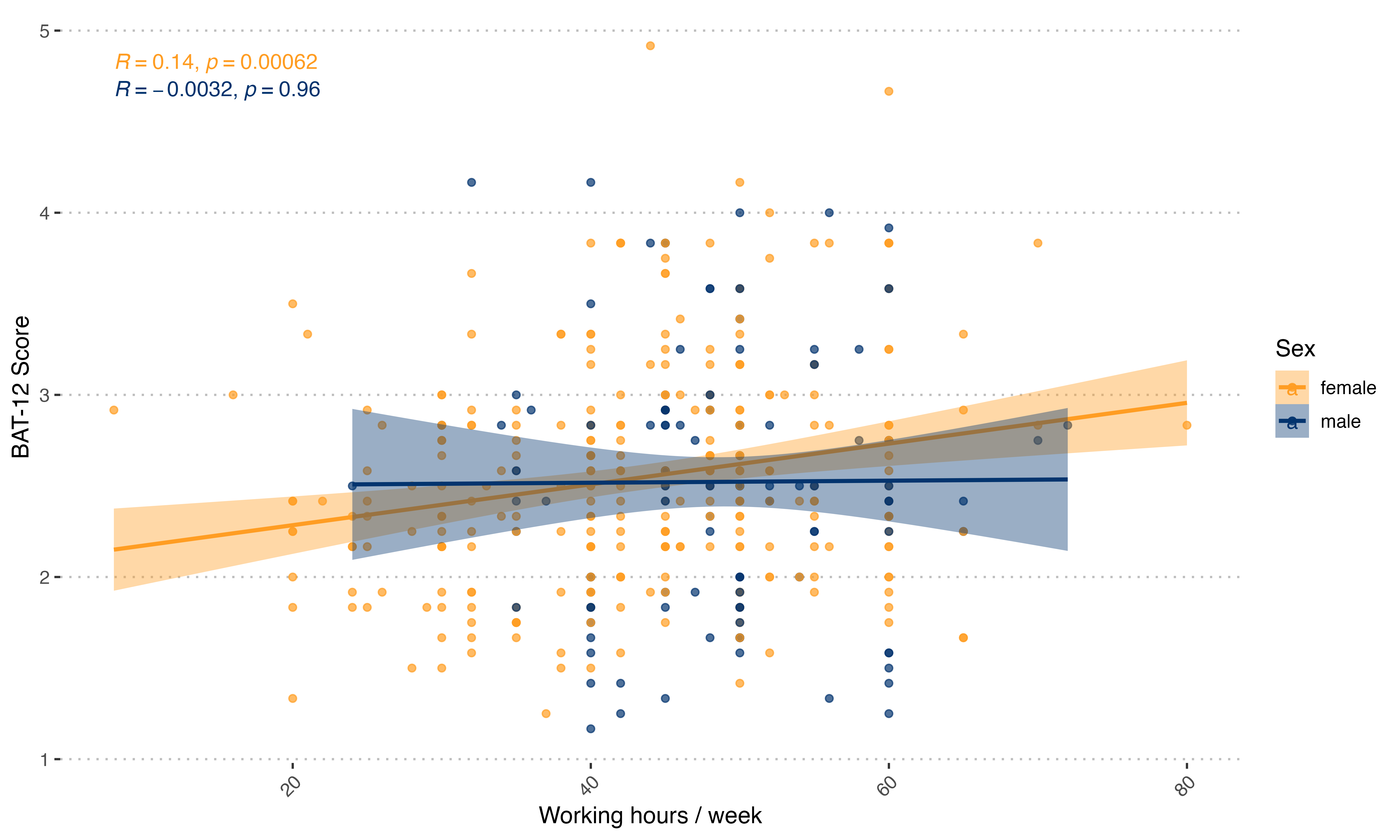


**Supplementary Table 2.** Debriefing offers by the employer, either local offers or structured offers following external protocols

|  | Residents, N = 282 | Specialists, N = 159 | p-value^1,2^ |
| --- | --- | --- | --- |
| **Debriefing offers**  Not available / existing  I don’t know  Local offer available  I didn’t use it  I already used it  CISM available  I didn’t use it  I already used it | 122 (43%)  133 (47%)  10 (4%)  9 (3%)  6 (2%)  2 (1%) | 68 (43%)  54 (34%)  7 (4%)  14 (8%)  13 (8%)  3 (2%) | 0.001** |
| ^1^ *Fisher’s exact test*, ^2^ *Correction for multiple testing using holm’s method, CISM: Critical incident stress management* | | | |

**Supplementary Table 3.** Multivariate model

| Category | Included Variables |
| --- | --- |
| **Ethical Factors** | - Ethical challenges in patient care decisions  - Disagreement about therapy goals  - Uncertainty about patient’s will  - Emotional burden from patient fates |
| **Team Communication** | - Poor communication within the team  - Poor communication with other departments  - Inconsistent instructions  - Fear of superiors |
| **Institutional Factors** | - Economic influence  - Overload due to patient numbers  - Overload due to unmanageable tasks  - Other institutional reasons |
| **Direct Assault** | - Experiencing direct assaults during work |
| **Mistakes** | - Errors made by team members  - Wrong decisions by team members  - Own mistakes |
| **Professional Skills** | - Gaps in knowledge  - Lack of practical skills |

**Supplementary Table 4.** Response to the question ‘Have you been prepared to deal with stressful events?’

|  | Total,  N = 445 | Residents,  N = 285 | Specialists,  N = 160 | p-value^1,2^ |
| --- | --- | --- | --- | --- |
| **I received preparation**  No preparation  Before university  As undergraduate  During residency  Training-independent | 279 (63%)  16 (4%)  77 (17%)  72 (16%)  76 (17%) | 178 (62%)  7 (2%)  55 (19%)  41 (14%)  42 (15%) | 101 (62%)  9 (6%)  22 (14%)  31 (19%)  34 (21%) | 0,92  0.44  0.46  0.46  0.44 |
| ^1^ *Fisher’s exact test*, ^2^ *Correction for multiple testing using Holm’s method* | | | | |

**Supplementary Figure 3.** Correlation matrix of possible confounding variables in multivariate analysis.


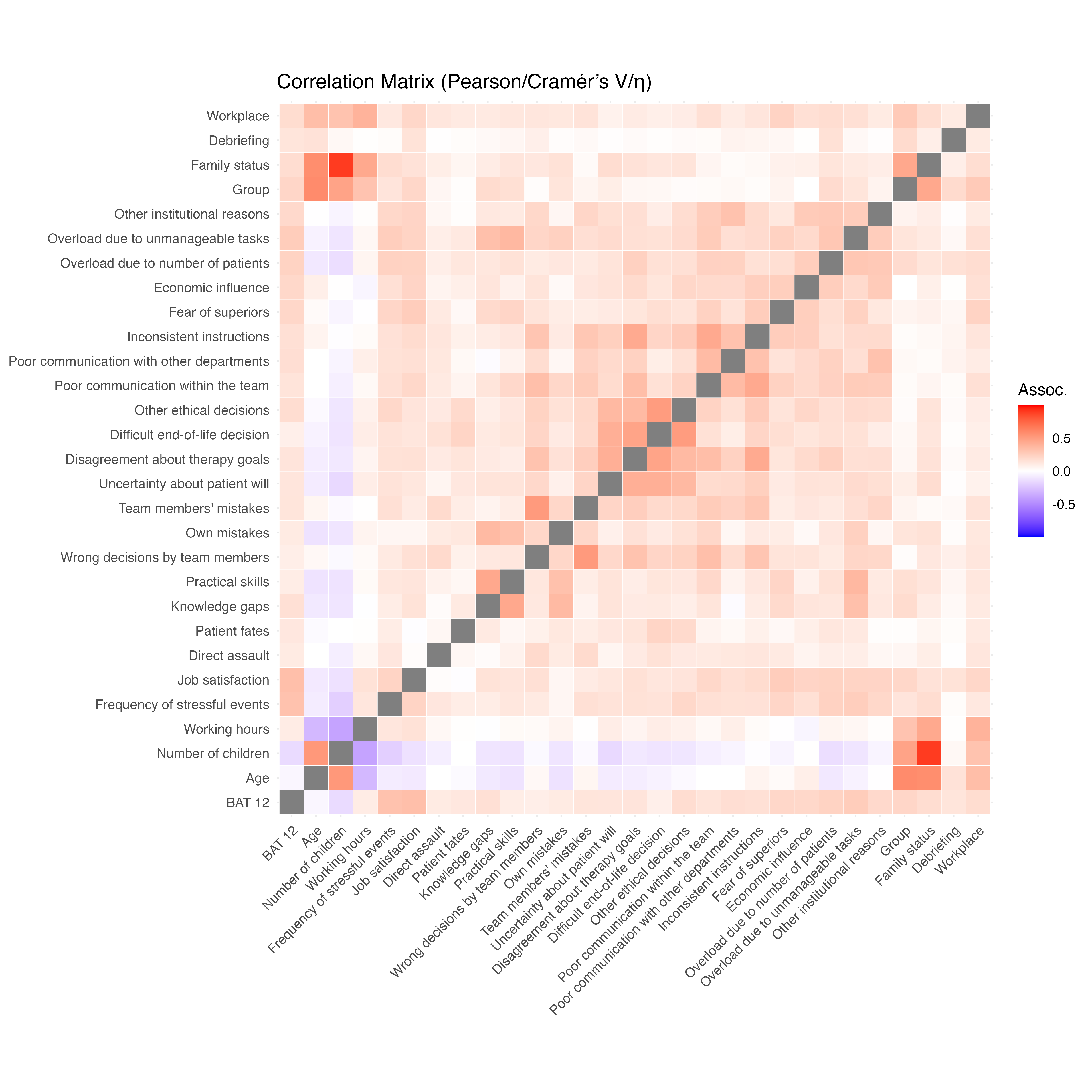


**Supplementary Figure 4.** Burnout levels (BAT-12) in different workplaces. Box plots show median ± IQR.


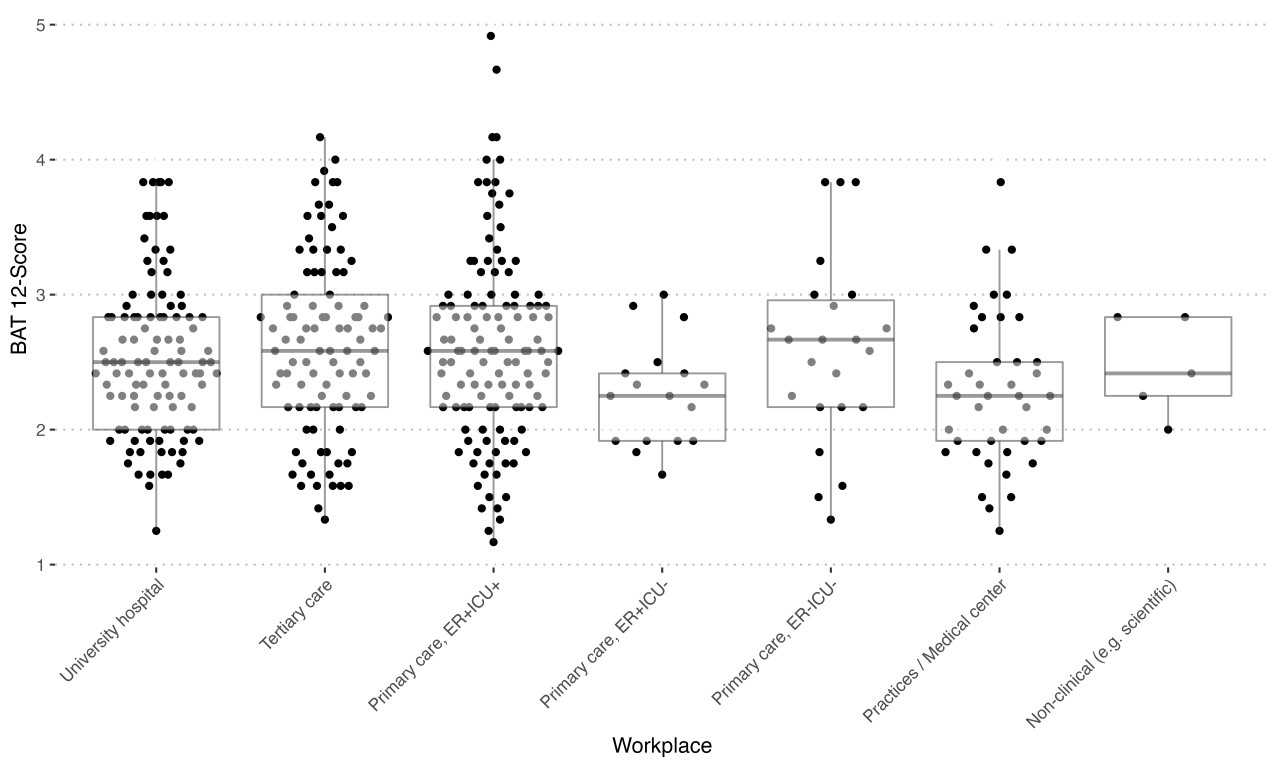


**Supplementary Figure 5.** Correlation of the item ‘I am generally satisfied with my current job’ (5 = agree, 1 = disagree) and burnout symptoms (BAT-12). Box plots show median ± IQR.


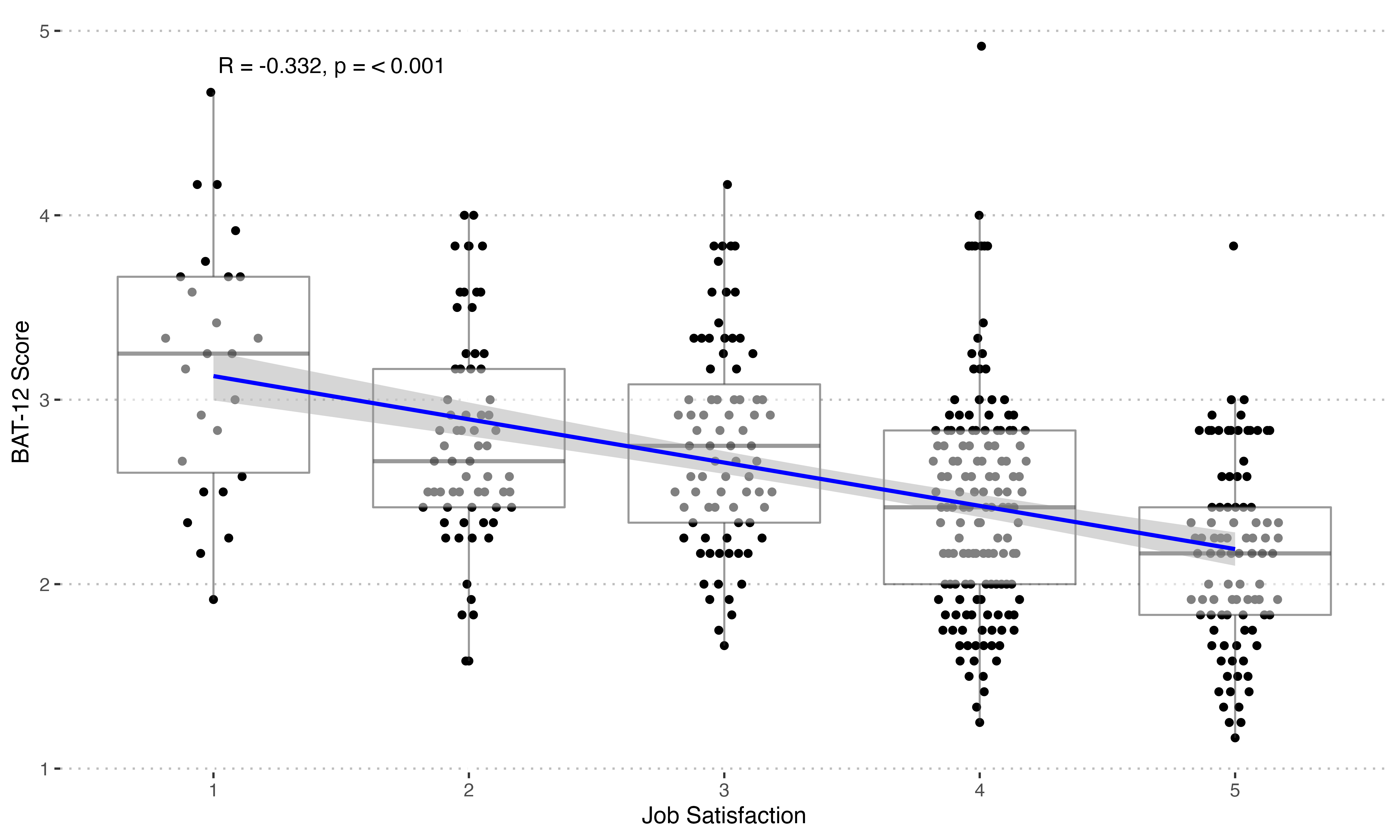


**Supplementary Figure 6.** Mosaic plot of the item ‘I am generally satisfied with my current job’ (5 = agree, 1 = disagree) in different workplaces.


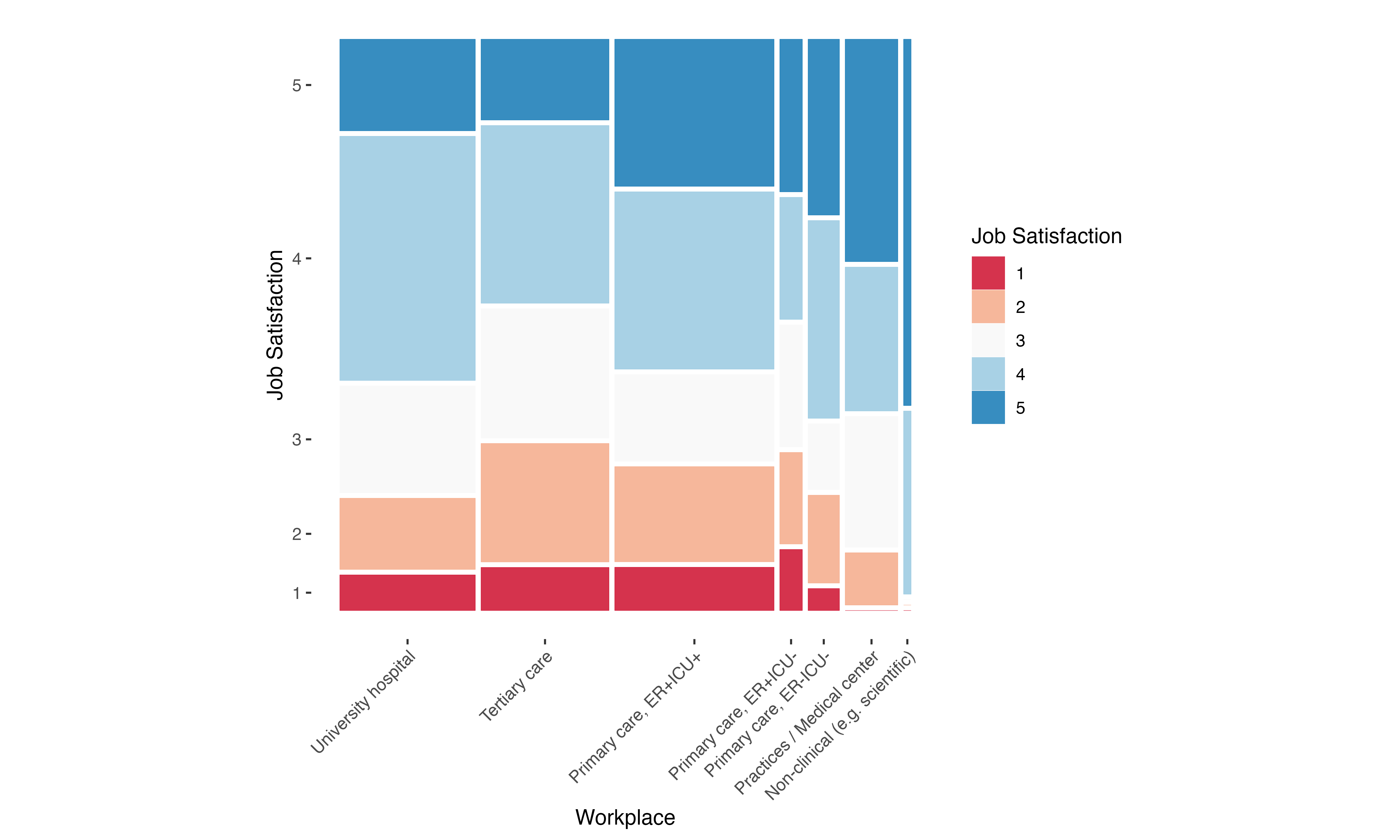

Supplement: Supplementary file 1 — Supplementary Material 1 [file 42466_2025_415_MOESM1_ESM.docx]
